# Prevalence and genotypes of Rotavirus among children under 5 years presenting with diarrhoea in Moshi, Tanzania: a hospital based cross sectional study

**DOI:** 10.1186/s13104-017-2883-3

**Published:** 2017-10-30

**Authors:** Deborah N. Mchaile, Rune N. Philemon, Sonia Kabika, Evelyn Albogast, Kikoti J. Morijo, Emmanuel Kifaro, Blandina T. Mmbaga

**Affiliations:** 1 Department of Pediatrics and Child Health, Kilimanjaro Christian Medical Centre, Moshi, Tanzania; 2 Kilimanjaro Christian Medical University College, Moshi, Tanzania; 3 Department of Veterinary Microbiology and Parasitology, Sokoine University of Agriculture (SUA), Morogoro, Tanzania; 4 Kilimanjaro Clinical Research Institute, Moshi, Tanzania

**Keywords:** Rotavirus, Diarrhoea, Under five, Moshi Municipality

## Abstract

**Background:**

Diarrhoea is a main cause of morbidity and mortality in children under 5 responsible for approximately four billion cases and 1.1 million deaths annually. In developing countries, it causes two million deaths each year. The major causative organism responsible is Rotavirus which is responsible for one-third of hospitalizations with approximately 40% mortality.

**Results:**

The prevalence of Rotavirus infection was 26.4% (73/277). The predominant strain of Rotavirus found was G1 21/73 (53.8%), followed by G8 9/73 (23.1%), G12 5/73 (12.8%), G9 3/73(7.7%) and G4 1/73 (2.6%). All serotypes identified were in children who had completed Rotavirus vaccination except for one who had G8 in whom the vaccine was introduced after they had completed immunizations.

**Conclusion:**

The overall prevalence of rotavirus has reduced from 33.2% in 2009 to 26.4% in 2016. We have found G1 to be the predominant serotype as well as other circulating serotypes namely G4, G8, G9 and G12. Despite a reduction in prevalence, there is a need for further rotavirus surveillance in the region.

**Electronic supplementary material:**

The online version of this article (10.1186/s13104-017-2883-3) contains supplementary material, which is available to authorized users.

## Background

Diarrhoea is a main cause of morbidity and mortality in children under 5 responsible for approximately four billion cases and 1.1 million deaths annually [1]. In developing countries, it causes two million deaths each year [2]. The major causative organism responsible is Rotavirus which is responsible for one-third of hospitalizations with approximately 40% mortality [3].

Rotavirus mainly affects children, with young children below five years being the most vulnerable. The prevalence of rotavirus is highest among children from 13 to 24 months (57.6%) followed by those less than 1 year (46.3%) and lowest among children aged 1 to 5 years [4].

Rotavirus is an RNA virus and a member of the Reoviridae family [5]. It has groups ranging from A to G with Group A having multiple strains and causing majority of childhood infections [6]. The Rotavirus has 14G and 11P serotypes which are critical to vaccine developments; the serotypes are the vaccine targets for stimulating a protective immune response [7]. G1P[8] is the commonest circulating serotype accounting for two-thirds of infection [8].Strain diversity has been reported and is greatest in Africa and Asia due to mixed infections and proximity to domestic animals shedding the virus [8]. There are also changes in genotypes circulating due to seasonal variation [9]. In Africa the most prevalent strains were G1P [8] and G3P [8, 10].

Rotavirus vaccine (*Rotarix*) was introduced into the Tanzania Expanded Programme of Immunization in January 2013. Studies have shown vaccine effectiveness to be 98% in high income countries as compared to 59% in middle income countries [11]. In Malawi data showed the vaccine to be 64% effective in rotavirus negative individuals [12]. In the pre-rotavirus vaccine era, rotavirus diarrhoea resulted in 220,000 annual hospital admissions, 1.8 million healthcare visits and 7.1 million episodes of diarrhoea among children living in developed countries [13]. In the United States, there were an estimated 20–60 deaths, 55,000 to 75,000 hospitalizations and 410,000 outpatient visits annually [14]. In the post vaccination era, it is estimated that approximately 36% of diarrhoea hospitalizations in children less than 5 years in countries where rotavirus surveillance is being carried out, can be attributed to rotavirus infection [3].

Prior to introduction of vaccine in Tanzania, the prevalence of Rotavirus in Dar-es-Salaam was 32.5% [15]. Previous studies have reported the emergence of new strains such as G9 and others like G12, G4 and G8 in Dar-es-Salaam [15]. Despite this there have not been any published studies to date looking at the circulating serotypes in the Kilimanjaro region both in the pre-or post-vaccine era. Because of this we fail to understand if the diarrhoea cases being observed are due to different virus strains or the same strain targeted by vaccine. We also fail to comment on the circulating serotypes. This study aimed to determine the prevalence of Rotavirus among children under 5 in Moshi, Tanzania post vaccination and the circulating serotypes in the region. Kilimanjaro Region is one of Tanzania’s 31 administrative regions. It has 7 districts with the regional capital being Moshi municipality. Moshi is situated on the lower slopes of Mount Kilimanjaro and covers about 59^2^ km (23^2^ m) with a population of 184,292 per the 2012 census.

## Methods

### Study design and setting

This was a hospital based cross sectional study involving children under 5 years of age in Moshi Municipality presenting with diarrhoea from January 2016 to May 2016.

Four health facilities were purposively selected to cover multiple tiers of health care and these included: St Joseph Hospital, Kilimanjaro Christian Medical Centre (KCMC) Hospital, Majengo and Pasua Health Centre. KCMC is one of the zonal referral hospitals in Tanzania; it serves as a referral, research and teaching hospital, located in the Northeastern part of Tanzania. It serves five regions in the Northern part of Tanzania, namely, Kilimanjaro, Tanga, Arusha, Manyara and Singida with an estimated population over 15,000,000 people. It has a bed capacity of 450 beds. St Joseph is a designated District Hospital which has several departments and admits approximately 1787 pediatric patients annually. Majengo Health Centre and Pasua Health Centre are secondary level health centres. Both serve an average of 4920 patients per year, (out-patient department), 564 in patients annually at Pasua Health Centre and 5491 outpatients annually at Majengo Health Centre.

### Study population

All children aged 2–59 months presenting with passage of 3 or more motions of loose or watery stools per 24 h receiving care in the four study centres during the study period were included. Sample size was estimated per hospital attendances. At least 17 patients were expected from each of the 4 study sites each month. Data was collected for 5 months. The expected sample size was 340.

### Inclusion and exclusion criteria

We included all children presenting with diarrhoea aged 2–59 months and excluded those children whose parents/guardian did not agree nor sign the consent form.

### Data collection and study variables

A questionnaire was used to collect socio-demographic and clinical data from participants with the aid of research assistants. Our outcome variable was rotavirus and predictor variables were age, sex, duration of diarrhoea, frequency of diarrhoea, completion of rotavirus vaccine and degree of dehydration.

The fecal specimens were collected by research assistants and transported to the department of clinical laboratory, molecular biology unit and stored at − 20 °C until used for the detection of group A rotavirus.

### Rotavirus Antigen Detection

The specimens were tested by a solid-phase sandwich type enzyme immunoassay method according to manufacturer instructions Rotavirus Antigen ELISA, Epitope diagnostic Inc (Carroll Road San Diego, USA), Catalogue No: KT 841. The optic density (OD) values above the cut off point 1.1 x (mean absorbance of negative control + 0.08) were considered positive for rotavirus antigen.

### Molecular typing of rotavirus

#### Viral RNA extraction

1 ml of 10% (w/v) fecal suspension in phosphate-buffered saline (PBS) was prepared by thorough mixed using vortex and clarified by centrifugation for 5 min at room temperature and 10,000 rpm. The clarified supernatant was collected for viral genome extraction. Double-stranded RNA was extracted according to the manufacturer’s instructions from 140 μLs of collected supernatant using the QIAamp Viral RNA Mini Kit (Qiagen/Westburg, Leusden, Netherlands), Catalogue Number: 52906.

#### Reverse Transcription and G-typing of Rotaviruses

About 40 μl of extracted nucleic acid were transferred to a PCR tube. The dsRNA was denatured at 97 °C for 5 min, and then chilled on ice for 2 min. The 10X buffer II (Invitrogen) 7.0 μl, 50 mM MgCl_2_ 7.0 μl, Random primers 1.0 μl, dNTPs (10 mM) 2.0 μl, M-MLV (200 U/μl) Invitrogen 2.0 μl, RNase-free H2O 11.0 μl, to make total volume of 30.0 μl. Then 30 μl of RT mix was added to each tube containing the extracted RNA. Incubated at 42 °C for 50 min, and then was incubate again at 95 °C for 5 min followed by chilling on ice for 2 min. The total volume was 70 μl of cDNA was obtained and stored at − 20 °C ready for PCR.

#### G-typing consensus PCR (VP7)

The first-round PCR mix was prepared with 10X buffer II (Invitrogen) 4.5 μl, 50 mM MgCl2 2.0 μl, dNTPs (10 mM) 1.0 μl, Taq polymerase (5 U/μl) (Invitrogen) 0.2 μl, Primer VP7-F (20 pmol/μl) 1.0 μl, Primer VP7-R (20 pmol/μl) 1.0 μl, RNase-free H2O 35.3 μl, to make total volume 45.0 μl. 45 μl of PCR mix was added to each PCR tube, and 5 μl of cDNA (from the RT reaction) was added, and tubes transferred to thermal cycler and cycle conditions as provided. The samples were then separated in 2% agarose gel electrophoresis to see positive samples.

The second-round PCR mix was then prepared with 10X buffer II 4.8 μl, 50 mM MgCl2 2.5 ul, dNTPs (10 mM) 1.0 ul, Taq polymerase (5 U/μl) 0.2 ul, Primer VP7-R (20 pmol/μl) 1.0 μl, and 1.0 μl (20 pmol/μl) of each strain specific primers G1 to G12, RNase-free H_2_O 30.5 µl to make total volume of 48.0 μl. 48 μl of second-round mix was then added to each PCR tube and then 2 μl of first round product was added, and PCR was run under the following temperature cycles conditions.

### Data analysis

Data was analysed using SPSS version 22. Descriptive summaries were prepared. Categorical and Numerical data were summarised using their respective measures of central tendency and measures of spread and presented using tables.

## Results

In total, 277 children under 5 years of age presenting with diarrhoea from our four study sites were enrolled during the study period. We failed to meet the estimated sample size due to low turnout of patients during the study period. Of these 16 were from Majengo Health Centre,70 from KCMC Hospital,117 from St Joseph District Hospital and 74 from Pasua Health Centre respectively. The median age was 11 (IQR 56) months with more than half being females 143/277 (51.6%). The majority reported to be living in Moshi urban 239/277 (86.3%) and reported tap water as their main source of water 257/277 (92.8%); only 84/277 (30.3%) caregivers reported to boil drinking water (Table 1).

**Table 1:** Social-demographic characteristics of participants (n = 277)

Characteristic	No	%
Age (months) (n = 270)*		
< 12	155	57.4
13–24	75	27.8
> 24	40	14.8
Median (IQR)	11 (56)	
Sex		
Male	134	48.4
Female	143	51.6
Residence		
Moshi urban	239	86.3
Moshi rural	22	7.9
Mwanga	5	1.8
Others	11	3.9
Water source		
River	5	1.8
Tap	257	92.8
Well	15	5.4
Drink boiled water		
Yes	84	30.3
No	193	69.7
Type of toilet used		
Bush	2	0.7
Flush toilet	159	57.4
Pit latrine	116	41.9
Rotavirus vaccine completed		
Yes	259	93.5
No**	18	6.5

We collected data from 277 children under 5 years of age presenting with diarrhoea

* Of these 3 did not specify a date of birth hence we had only 273 children in the age category

** 6 of the children had not been vaccinated as the vaccine was introduced after their time

The overall prevalence of Rotavirus in our study was 26.4% (73/277); 29/73 (39.7%) in children aged less than 12 months, 34.2% (25/73) in children aged 13-24 months and 21.9% (16/73) among children older than 24 months.

Of those with Rotavirus positive by ELISA, 41/73 (56%) had PCR done for G-typing consensus PCR (VP7) using strain specific primers. PCR was performed on 41 samples out of 73 due to reagent stock out. The most common serotypes were G1 21/39 (53.8%) followed by G8 9/39 (23.1%) and G12 5/39 (12.8%), (Table 2). Only one child with G8 serotypes had not received Rotavirus vaccination since the vaccine had not been introduced in the Tanzanian EPI schedule during the time he was young, all others with G serotype identified had received the vaccine.

**Table 2:** G serotypes in patients with Rotavirus (n = 41)

Genotype	n*	%	Completed rotavirus vaccine
G1	21	53.8	21/21
G4	1	2.6	1/1
G8	9	23.1	8/9
G9	3	7.7	3/3
G12	5	12.8	5/5

G genotyping in children who were positive for Rotavirus by ELISA

*We did genotyping on 41 samples, though two failed testing hence only 39 were included in the final analysis

## Discussion

This study focused on determining the prevalence and genotypes of Rotavirus among children under five presenting with diarrhoea in four hospitals/health centres in Moshi Municipality in Northern Tanzania. We found the prevalence of Rotavirus to be 26.4% with children less than 24 months of age being most affected. The genotypes seen in children who had Rotavirus were G1, G4, G8, G9 and G12. Those who had completed Rotavirus vaccine had G1 serotype but also displayed the other strains.

In comparison to other studies done around the world; our findings were similar to studies done in Mali by Ouermi et al. [16] and in Tanzania, Mwanza by Temu et al. [17] which found prevalence’s of 22.7 and 20.7%, respectively. This similarity could be attributed to the fact that they had the same study duration and both were carried out in hospital settings. The former studies were both pre-vaccine studies which were carried out in Africa. The low prevalence in Mali could be due to their small sample size. Similarities were also seen in developed countries, in Japan a study done by Phan et al. [18] found a prevalence of 14.9%.

Differences were observed in Yemen in 2014 in a study done by Badani et al. [19] with a prevalence of 45.9% and in 2010 Nakawesi et al. [20] in Uganda found a prevalence of 45.4%. Both studies were hospital based and done before the Rotavirus vaccine was introduced in their respective regions. Gachanja et al. [21] in 2016 in Arusha, Tanzania reported a prevalence of 37% which is higher compare to the observed from our study. This study was done after introduction of Rotavirus vaccine as our study, however, the higher prevalence reported could be attributed by the fact that Arusha is a mainly pastoral community and living near livestock is a risk factor for acquiring Rotavirus as there are also bovine strains which can affect humans.

In our study we found the genotypes in 41 of the children who had Rotavirus were G1, G4, G8, G9 and G12.We also found genotype G1, G4, G9 and G12 was in all the children who had completed Rotavirus vaccine, except G8 where one child was not vaccinated. Our findings were similar to those by Kiulia et al. [22], 2008 in Kenya who found G1, G8 and G9 to be the circulating strains. This similarity could be attributed to both studies being done in the same geographical setting and same study population despite the former study being a pre-vaccine study. Both Moyo et al. [15] in Tanzania in (2014) and Ansari et al. [23] in Nepal in (2013) found genotypes similar to ours. This similarity could be attributed to the fact that the study included all children under 5 like ours and are known to be high risk group for acquiring Rotavirus as well as having used similar detection methods. Moyo et al. [15] in Tanzania was the first to describe pre-vaccine serotypes. Our study is of value as it’s the first to be done in the region. We cannot comment if the prevalence of these strains has increased or not since this is the first study to be done looking at the strain patterns in Kilimanjaro region. We now have baseline information to monitor trend and pattern these other subtypes.

Differences were noted in studies by Badani et al. [19] from Yemen in 2014, Sanchez-Uribe et al. [24], 2014 from Mexico who found G2P [4] and G9P [4]. This difference is due to the fact that both studies were able to do P genotyping unlike our study hence were able to report combination genotypes that existed in their respective region. Both G and P typing are critical for vaccine development [6]. Li et al. [25], 2014 in China found G1, G2, G3 and G9 and in which some strains were different from our study. This difference could be attributed to the fact that his study was done in the pre-vaccine era and hence no baseline serotypes were known making comparability difficult. Moyo et al. in 2007 [26] in Tanzania reported G1, G3 and G9 to be the serotypes presented with G1 being dominated which is similar to this study; however I did not find G3 in this study. This difference could be due to the fact it was a pre-vaccine study and done only in one season hence no room for changing of serotypes due to selection pressure exerted by vaccine or the seasonal effect of Rotavirus. However, looking at Moyo et al. study and our study as both done in Tanzania, we can say that majority of pre-and post vaccine strains are still dominating in Tanzania.

### Strengths and weaknesses

This is the first post vaccine study to be done in the Kilimanjaro region. It covered multiple tiers of health care and it’s the first to report genotypes in the Kilimanjaro region. We failed to meet the required sample size due to low turnout of patients during the study period and due to short duration of study also could not comment on seasonality of Rotavirus. We were not able to perform P-typing of Rotavirus in our study and were also not able to do DNA sequencing to check for similarities between the vaccine strain and the G1 we found. This was also a hospital based study so we may have missed a lot of non-hospitalised cases.

## Conclusion

Rotavirus is responsible for severe gastroenteritis in children under 5 years with a prevalence of 26.4% post vaccine era in Moshi, Tanzania. Most affected age group being children under 24 months of age. The dominated genotype was G1 53.8% with all children with this strain being received vaccine. Strain diversity was noted mostly in children who had completed rotavirus vaccine.

There is a need for continuous surveillance by relevant authorities of circulating serotypes as well further studies on vaccine efficacy. Long term assessment of vaccine potency, efficacy and circulating strain is needed so as to inform on the need for new vaccine or introduction of other genotype vaccines in the vaccination program.

## Additional file

**Additional file 1.** The questionnaire used for data collection from parents of children under 5 years of age presenting with diarrhoea in Moshi, Tanzania.

## Abbreviations

DNA: deoxyribonucleic acid
KCMC: Kilimanjaro Christian Medical Centre
PCR: Polymerase chain reaction
RNA: ribonucleic acid
SPSS: Statistical Package for the Social Sciences
