# Supplementary material for: Prevalence and genotypes of Rotavirus among children under 5 years presenting with diarrhoea in Moshi, Tanzania: a hospital based cross sectional study
Source: BMC Res Notes. 2017 Oct 30;10:542. doi: 10.1186/s13104-017-2883-3 (PMC5661935; doi:10.1186/s13104-017-2883-3)
Supplement: Supplementary file 1 — Additional file 1. The questionnaire used for data collection from parents of children under 5 years of age presenting with diarrhoea in Moshi, Tanzania. [file 13104_2017_2883_MOESM1_ESM.doc]

# QUESTIONNAIRE

## Hospital: _____________________________

**Date seen: ____/_______/20_____ (day/month/year)**

**Patient Information**

Patient Hospital Number ________________________________

Study ID number: ______________ Patient initials: _______________

Residence: ________________ Date of birth: _________________

Sex: Male___ Female___

**Clinical information:**

Presenting history:

1. Diarrhea: duration of symptoms: ________ days,

No. of episodes/past 24 hrs________

2. Vomiting Yes/No

If yes: Duration (days) ________

No. of episodes/ past 24 hrs _________

3. Fever: Yes/No

If yes: Duration (days) __________

4. Antibiotic treatment in the past 2 weeks; Yes/No

If yes which antibiotic and duration _________________________________

**Management of diarrhea at home**

5. When you noticed the child had diarrhea, what did you do? (Open ended question)

__________________________________________________________

6. Was the child given antibiotics to stop diarrhea? Y/N (If Yes which?)

__________________________________________________________

7. Was the child given traditional medicines to stop diarrhea? Y/N (If Yes which?)

______________________________________________________________________

8. Was the child given homemade solution? Y/N (If Yes, how did she/he prepare it)

__________________________________________________________________

9. Was the child given ORS? Y/N _________________________

10. Is the child still breastfeeding Y/N___________________?

Did he/she stop to breastfeed when diarrhea started? Yes/No

**Immunization history**

11. All immunizations received Yes/No

12. Rotavirus received Yes/No

13. Date of last Rotavirus immunization____**______________**

**Past medical history**:

14. Past episodes of diarrhea Yes/No

If Yes at what age: ____________months

**Nutritional history**

15. Breast feeding (< 2 years): Yes/No

If not breast feeding: Reasons: ____________________________

16. Weaning history: when started prior to current diarrhea episode:

1- 2 weeks: ____________

2 -4 weeks: ____________

Never done: _________

17. What diet is the child currently on?

___________________________________________

**Family History**:

18. Other family members with diarrhea Yes/No

If yes, who__________________________

19. Parents occupation: Father: ______________

Mother: ______________

20. Usual caretaker at home: Mother____, House help________, Other_____

21. Any known cases with diarrhea in the nearby houses: Yes/No

22. Number of people in the household? ______________________________________

23. Number of siblings_______________________________________

24. Number of siblings under 5_______________________________________

25. Toilet (type) ___________________________________

26. Water source __________________________________

27. Drinking boiled Yes/No

**Clinical examination:**

28. Temperature___________ ^0^C

29. Dehydration Yes/No

If yes; severe ( ) some ( ) no ( )

30. Treatment given Oral Rehydration Therapy: ( ).

Intravenous Fluid: ( )

31. HIV status Positive ( ), Negative ( ), Unknown ( )

**PERSON WHO COMPLETED THE FORM**

**Name: _____________________ Signature___________ Tel No. ____________**

**Date report completed: ----/----/----- (day/month/year)**
